# Supplementary material for: Structural basis of sequence-specific cytosine deamination by double-stranded DNA deaminase toxin DddA
Source: Nat Struct Mol Biol. 2023 Jul 17;30(8):1153–9. doi: 10.1038/s41594-023-01034-3 (PMC10442228; doi:10.1038/s41594-023-01034-3)

nature structural & molecular biology

Article

https://doi.org/10.1038/s41594-023-01034-3

Structural basis of sequence-specific cytosine deamination by double-stranded DNA deaminase toxin DddA

In the format provided by the authors and unedited

H1345C, F1375A

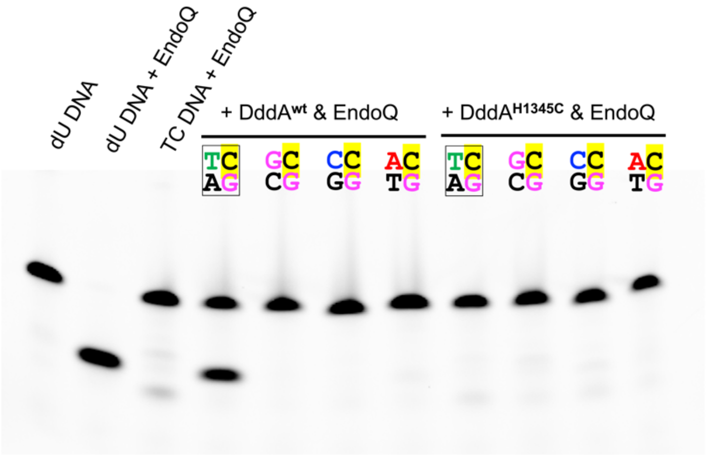

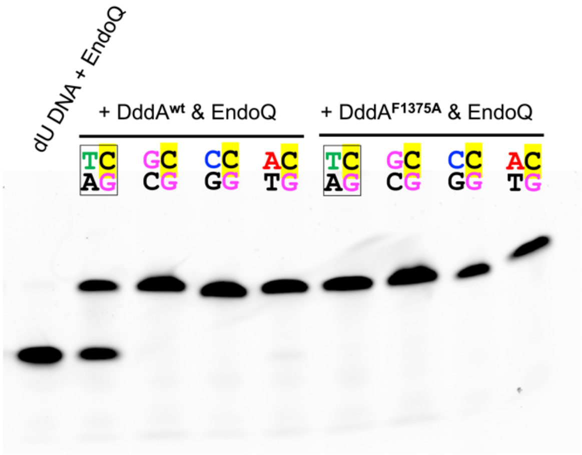

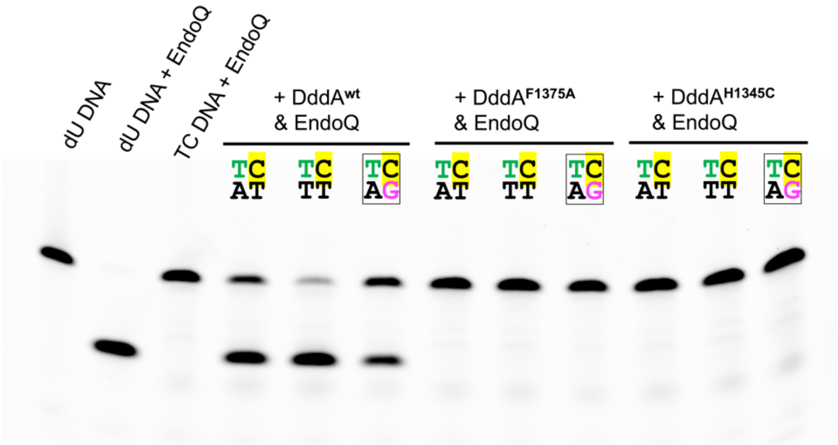

M1379R, F1375R

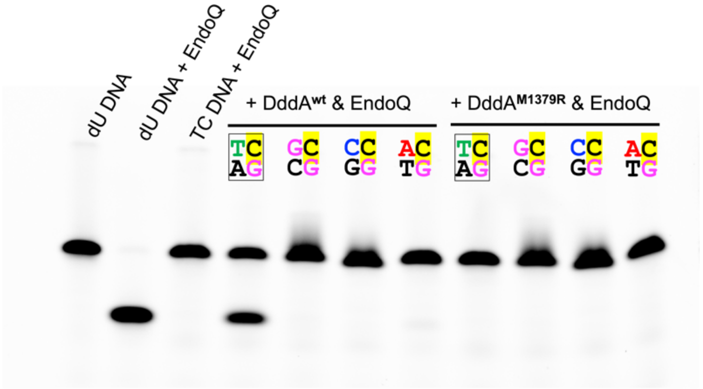

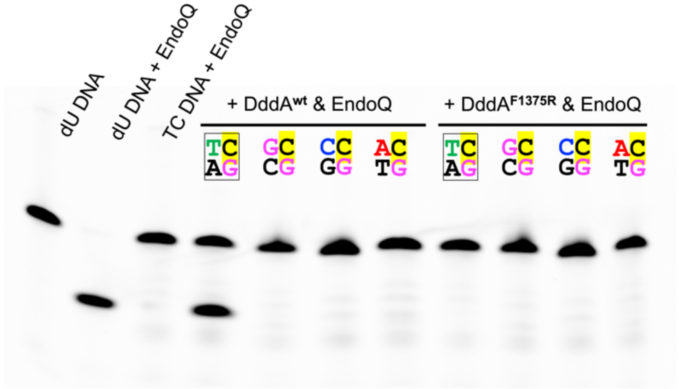

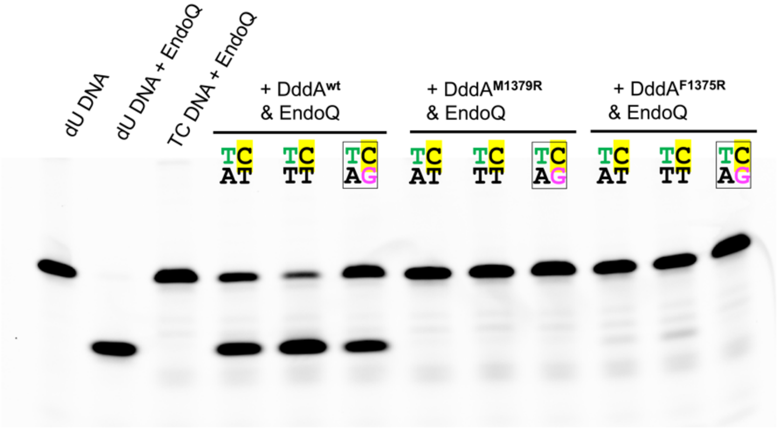

A1341T, A1341P

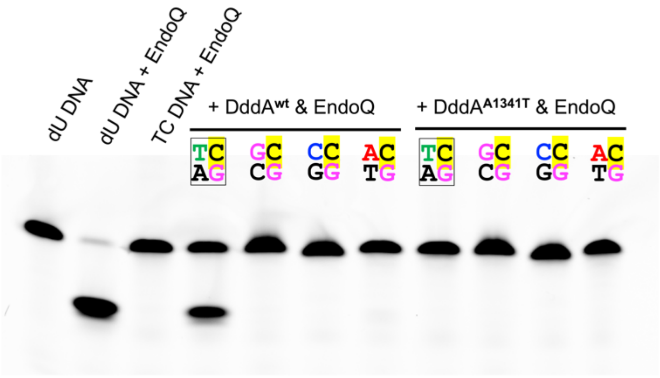

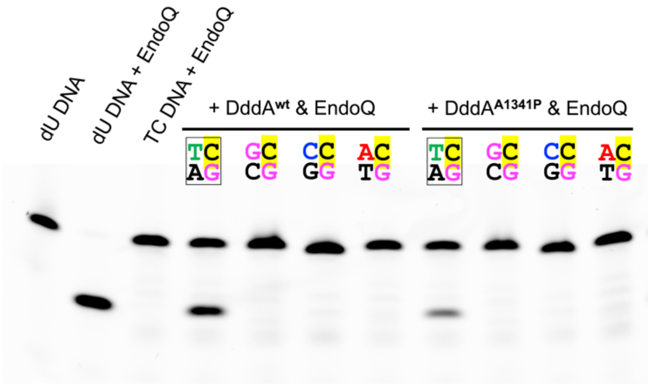

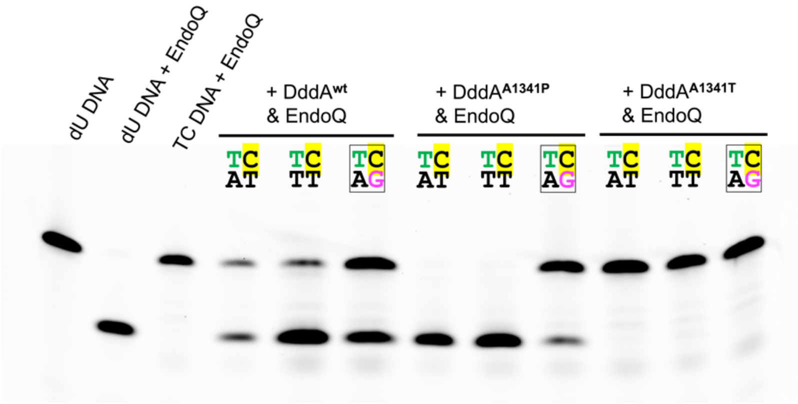

A1341Y, A1341E

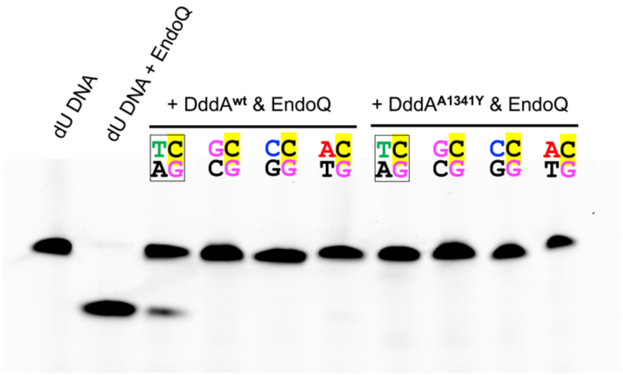

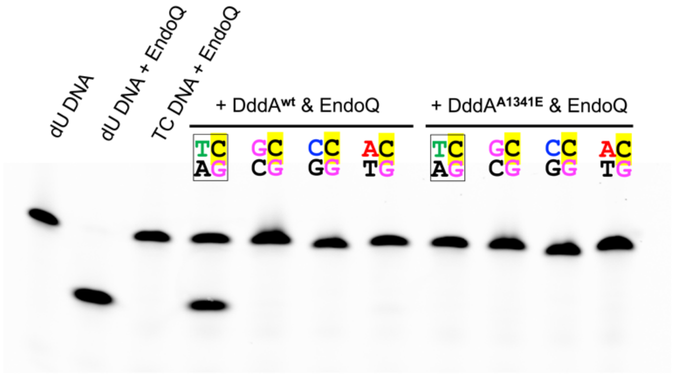

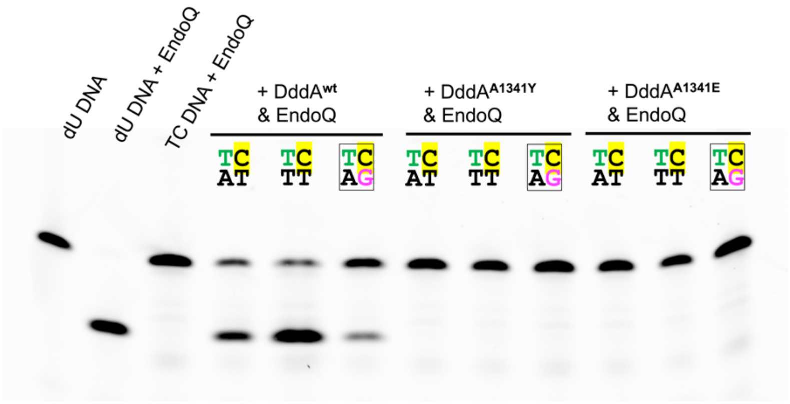

A1341S

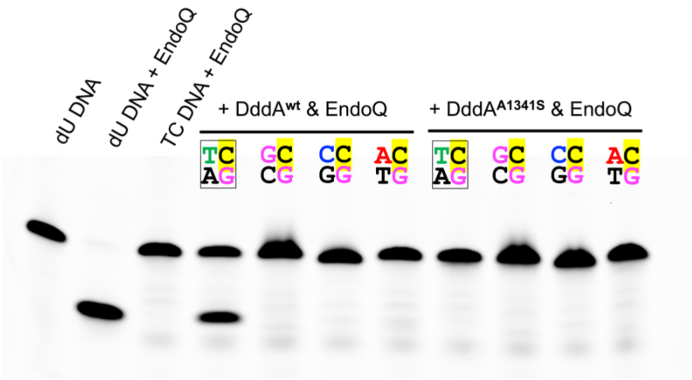
dU DNA
dU DNA + EndoQ
TC DNA + EndoQ
+ DddA^{wt} & EndoQ
+ DddA^{A1341S} & EndoQ
TC AG
GC CG
CC GG
AC TG
TC AG
GC CG
CC GG
AC TG

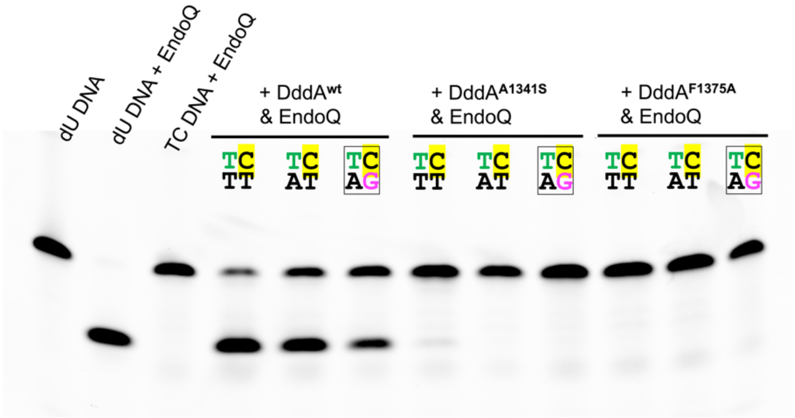
dU DNA
dU DNA + EndoQ
TC DNA + EndoQ
+ DddA^{wt} & EndoQ
+ DddA^{A1341S} & EndoQ
+ DddA^{F1375A} & EndoQ
TC TT
TC AT
TC AG
TC TT
TC AT
TC AG
TC TT
TC AT
TC AG

F1375Y

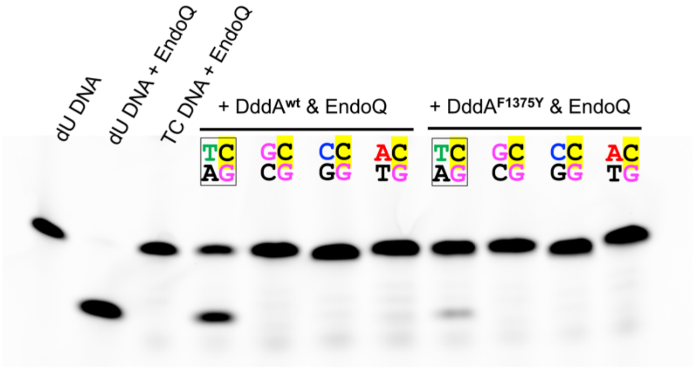
dU DNA
dU DNA + EndoQ
TC DNA + EndoQ
+ DddA^{wt} & EndoQ
+ DddA^{F1375Y} & EndoQ
TC AG
GC CG
CC GG
AC TG
TC AG
GC CG
CC GG
AC TG

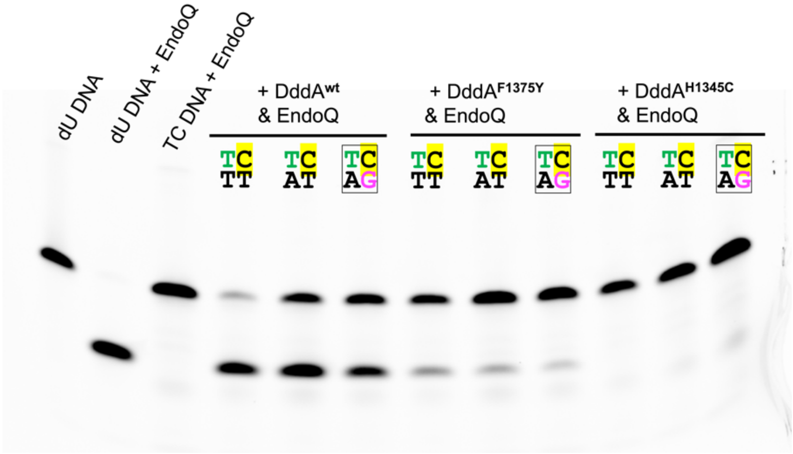
dU DNA
dU DNA + EndoQ
TC DNA + EndoQ
+ DddA^{wt} & EndoQ
+ DddA^{F1375Y} & EndoQ
+ DddA^{H1345C} & EndoQ
TC TT
TC AT
TC AG
TC TT
TC AT
TC AG
TC TT
TC AT
TC AG

M1379A

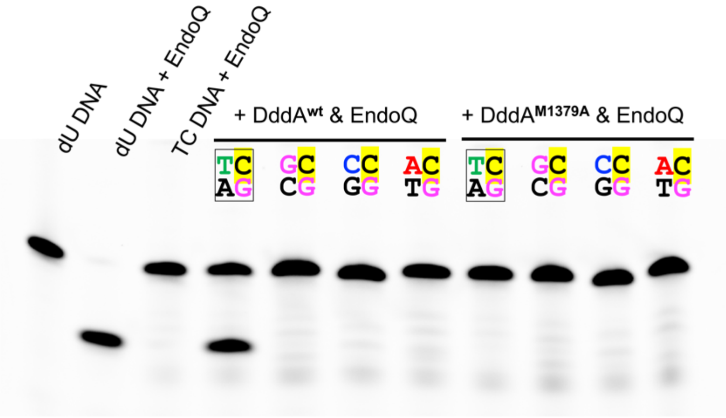
dU DNA
dU DNA + EndoQ
TC DNA + EndoQ
+ DddAwt & EndoQ
+ DddAM1379A & EndoQ
TC AG
GC CG
CC GG
AC TG
TC AG
GC CG
CC GG
AC TG

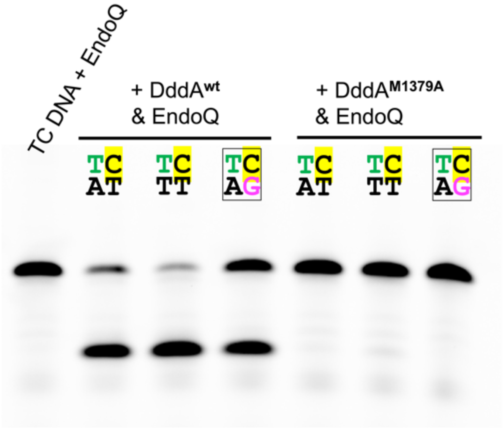
TC DNA + EndoQ
+ DddAwt & EndoQ
+ DddAM1379A & EndoQ
TC AT
TC TT
TC AG
TC AT
TC TT
TC AG

E1370K

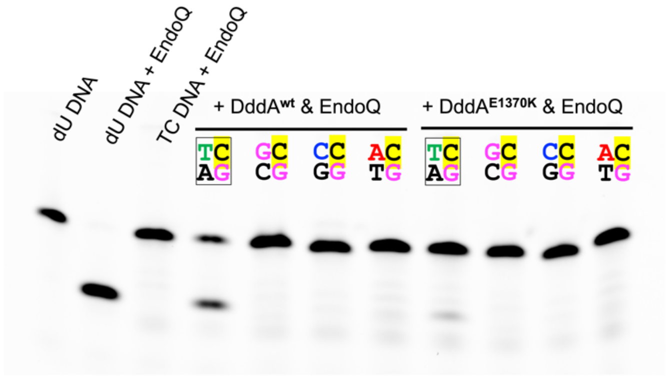
dU DNA
dU DNA + EndoQ
TC DNA + EndoQ
+ DddAwt & EndoQ
+ DddAE1370K & EndoQ
TC AG
GC CG
CC GG
AC TG
TC AG
GC CG
CC GG
AC TG

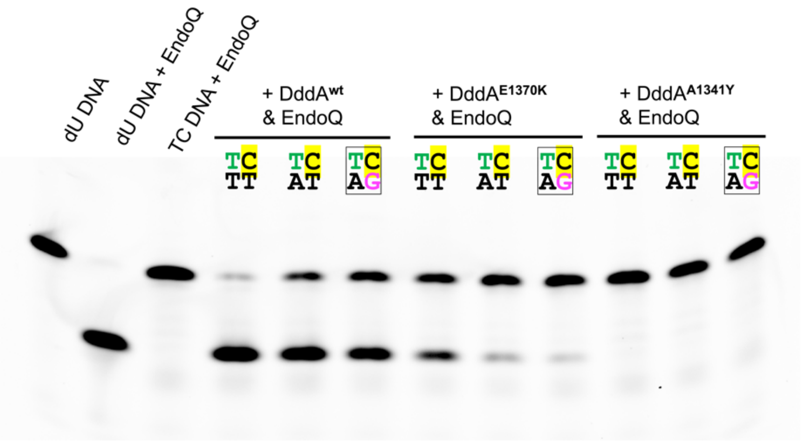
dU DNA
dU DNA + EndoQ
TC DNA + EndoQ
+ DddAwt & EndoQ
+ DddAE1370K & EndoQ
+ DddAA1341Y & EndoQ
TC TT
TC AT
TC AG
TC TT
TC AT
TC AG
TC TT
TC AT
TC AG

E1370R

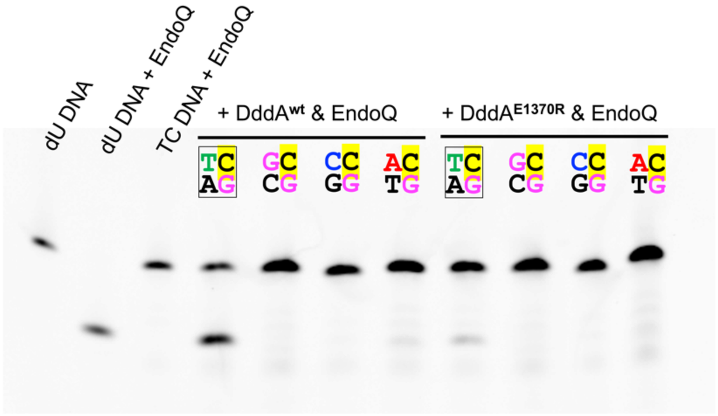

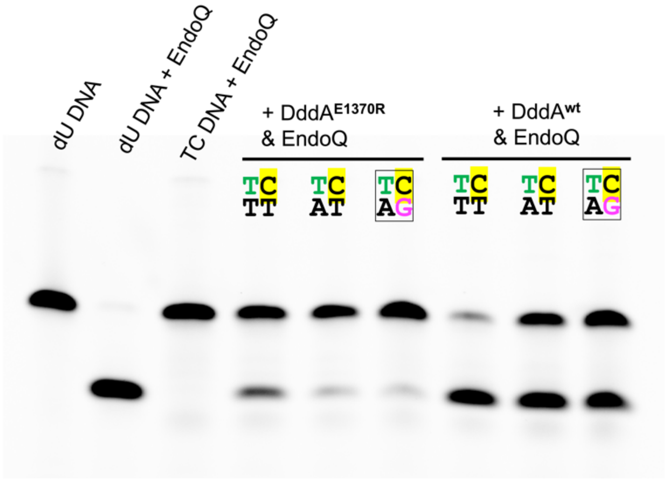

Supplementary Fig. 1 | Activities of various DddA mutants

The top and bottom strand sequences for the –1 and 0th positions are shown above each lane. Gels are representative of two replicates. Uncropped gel images are shown below.

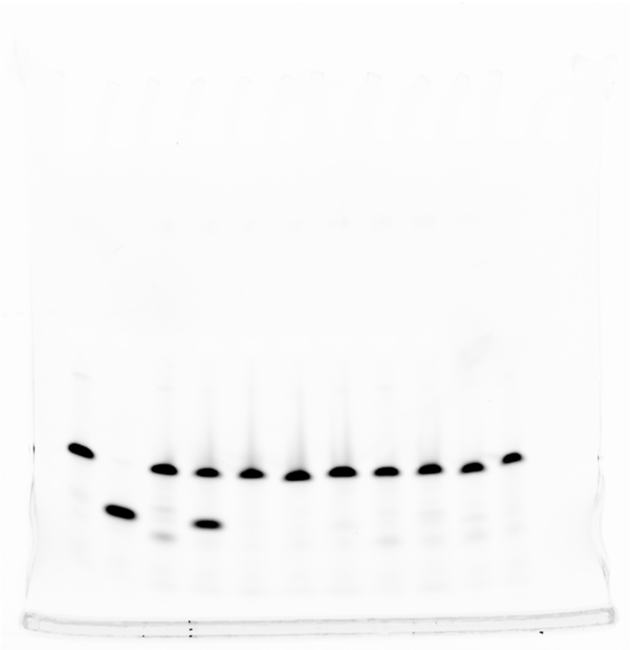

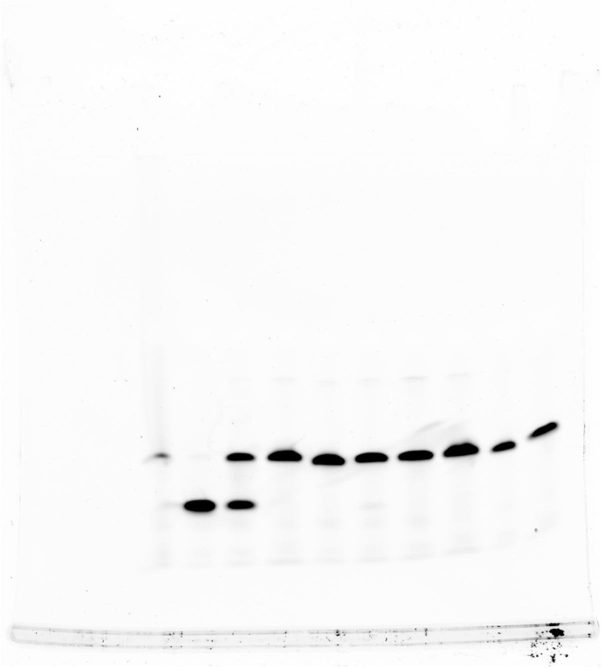

Uncropped gels
H1345C, F1375A

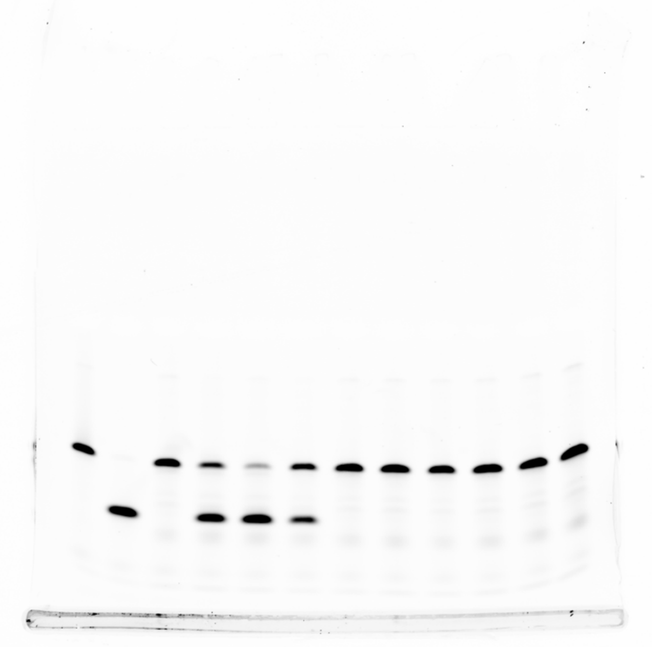

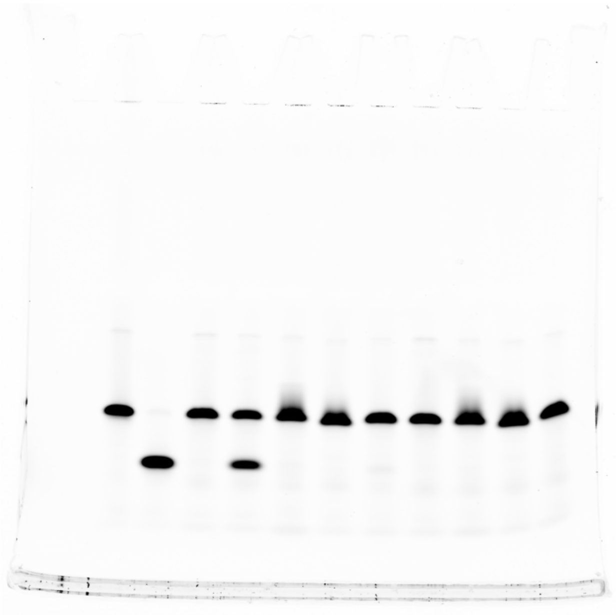

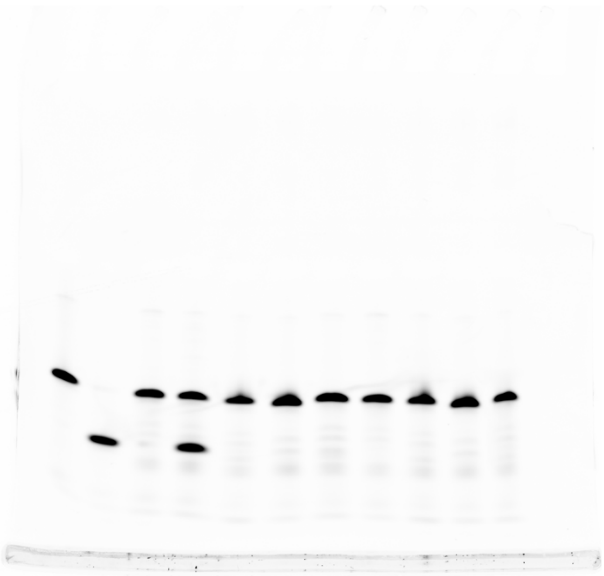

Uncropped gels
M1379R, F1375R

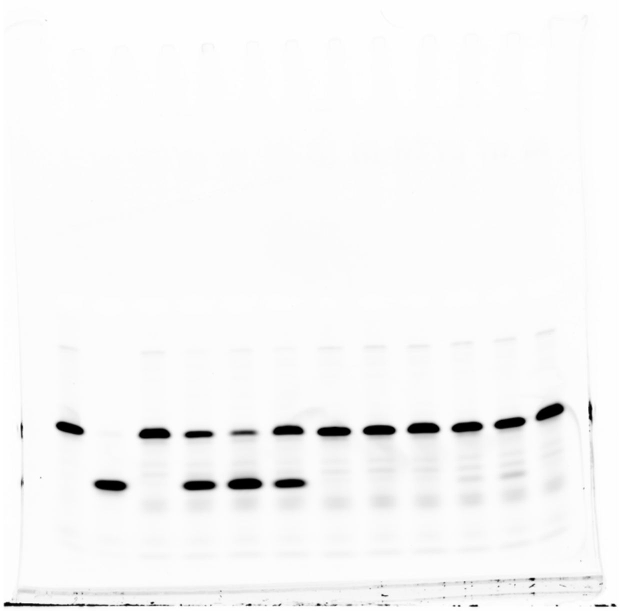

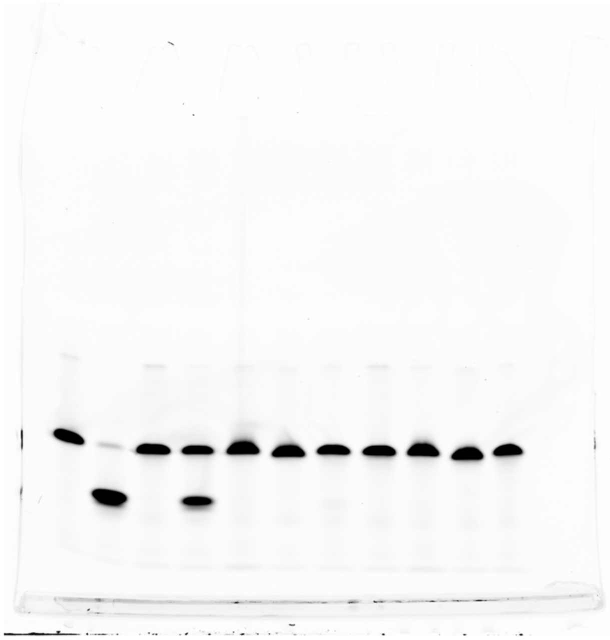

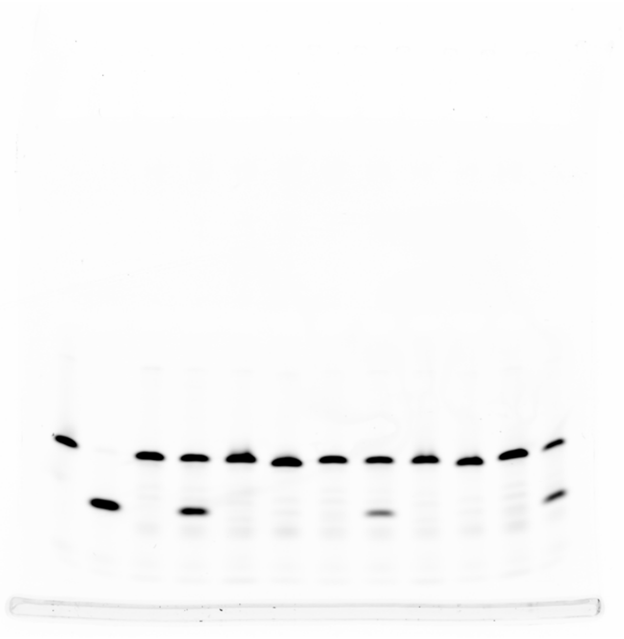

Uncropped gels
A1341T, A1341P

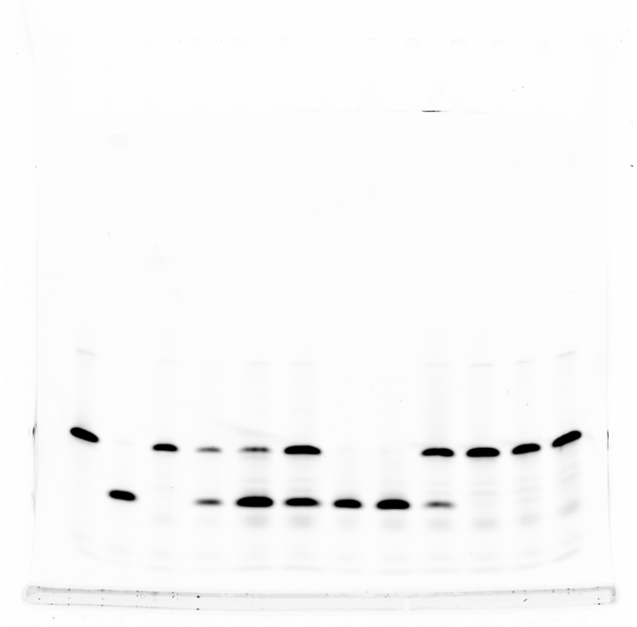

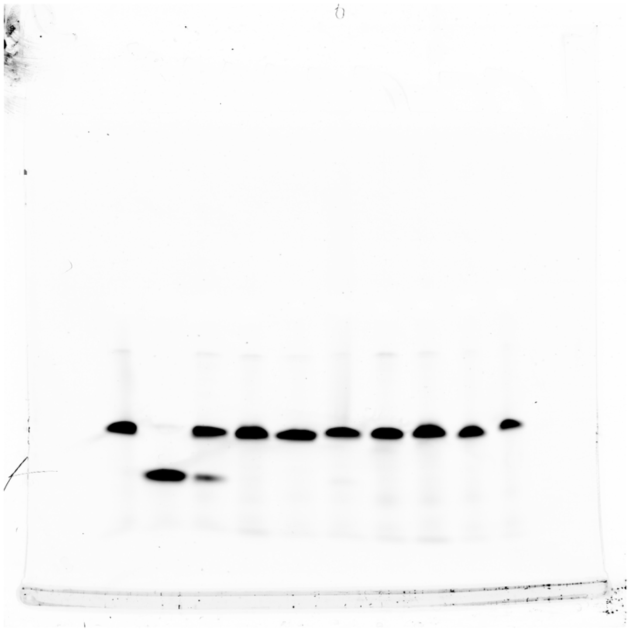

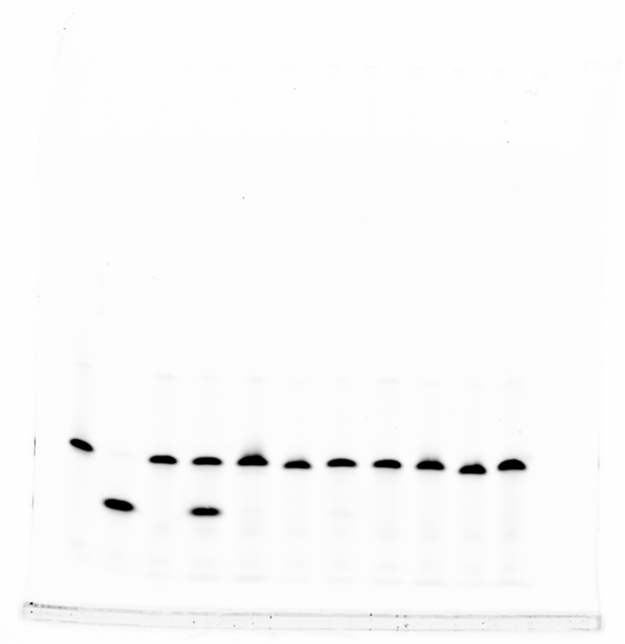

Uncropped gels
A1341Y, A1341E

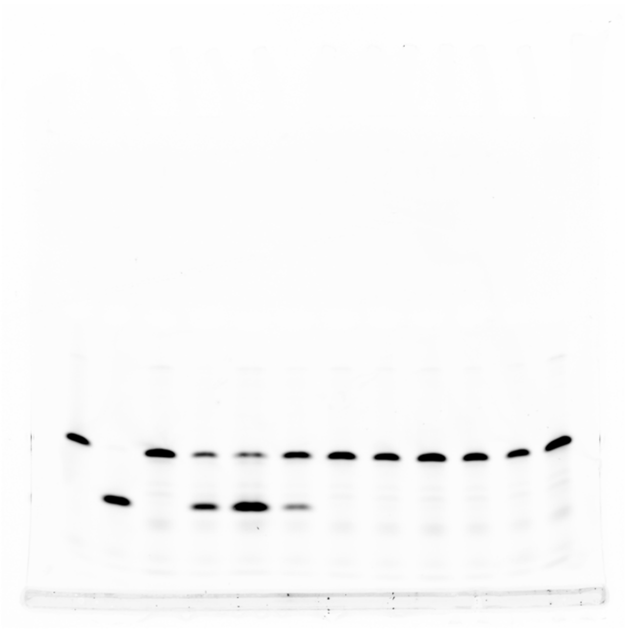

Uncropped gels
A1341S

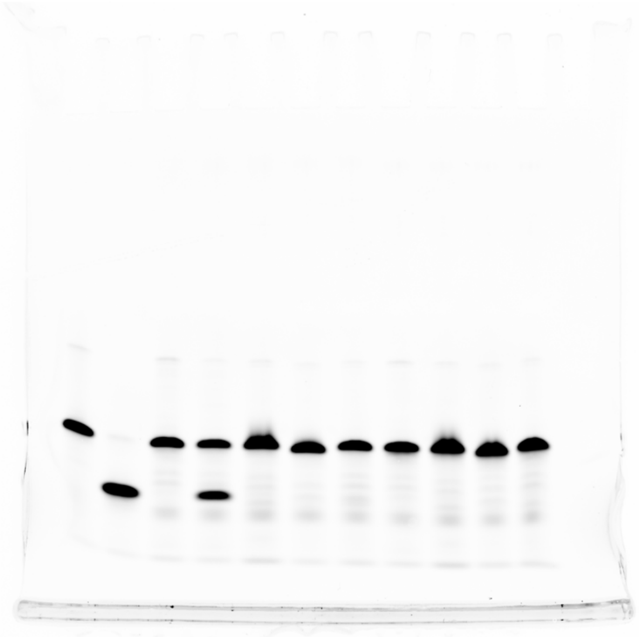

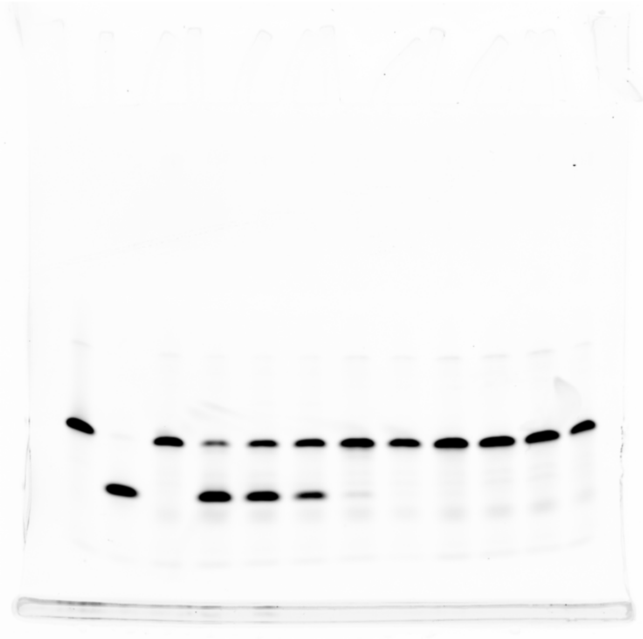

Uncropped gels
F1375Y

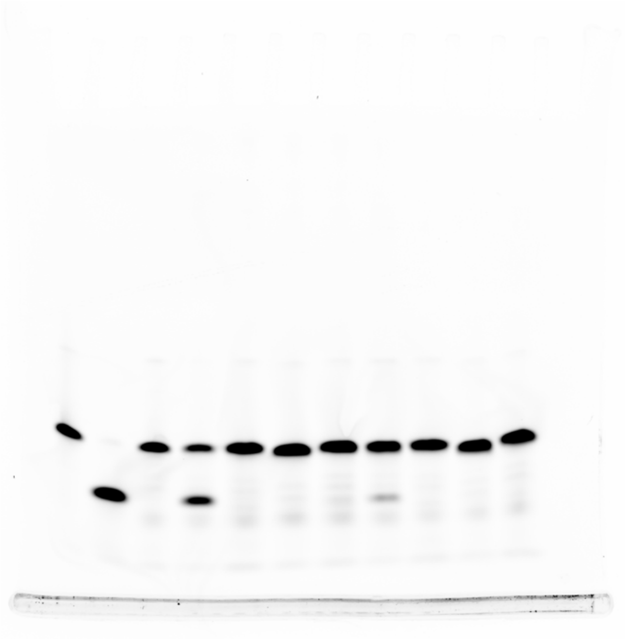

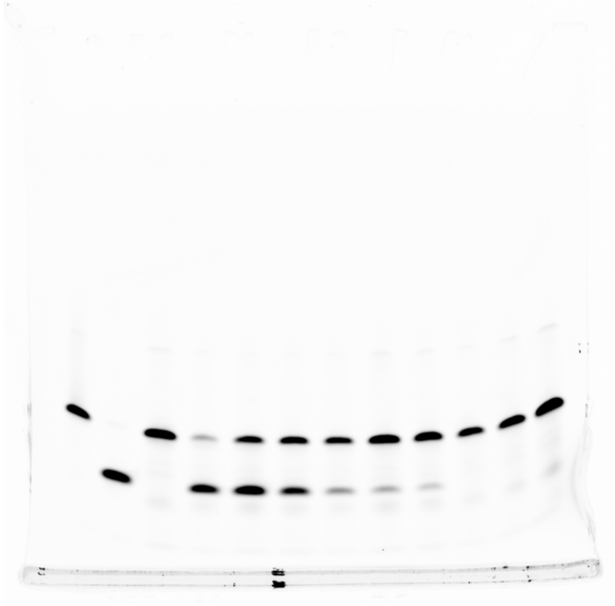

Uncropped gels
M1379A

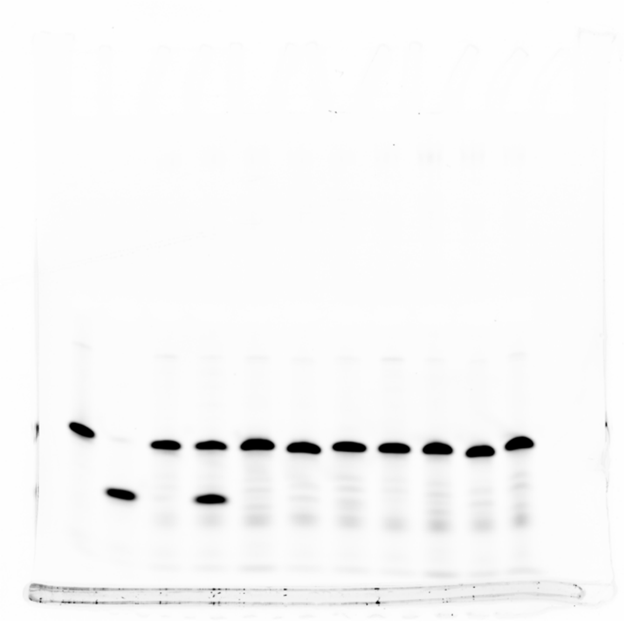

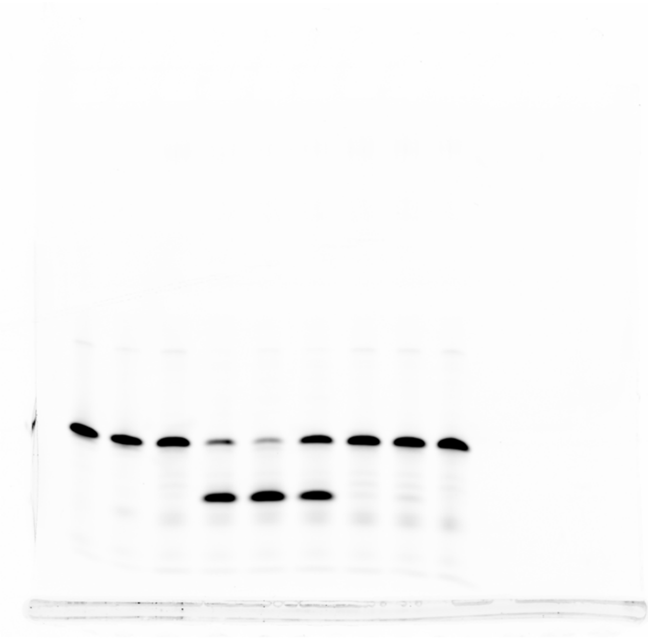

Uncropped gels
E1370K

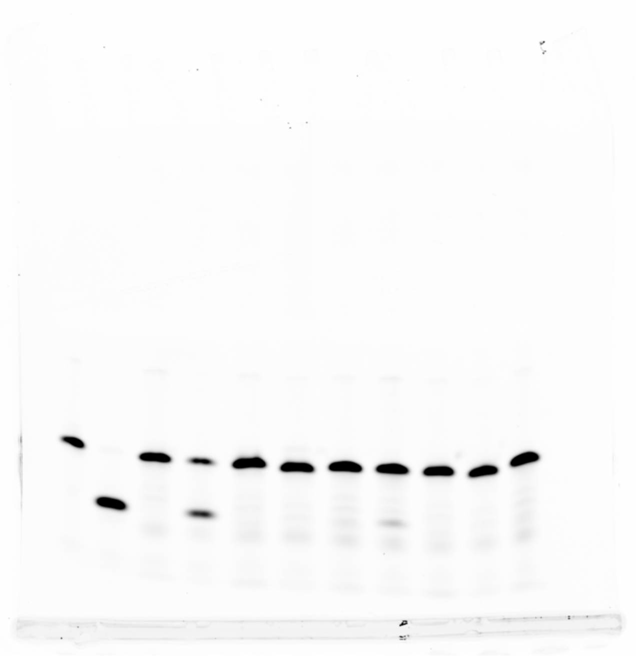

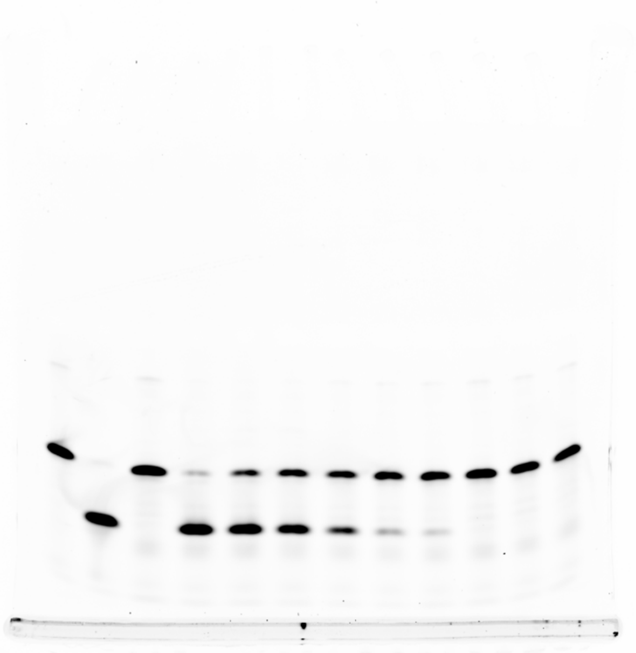

Uncropped gels
E1370R

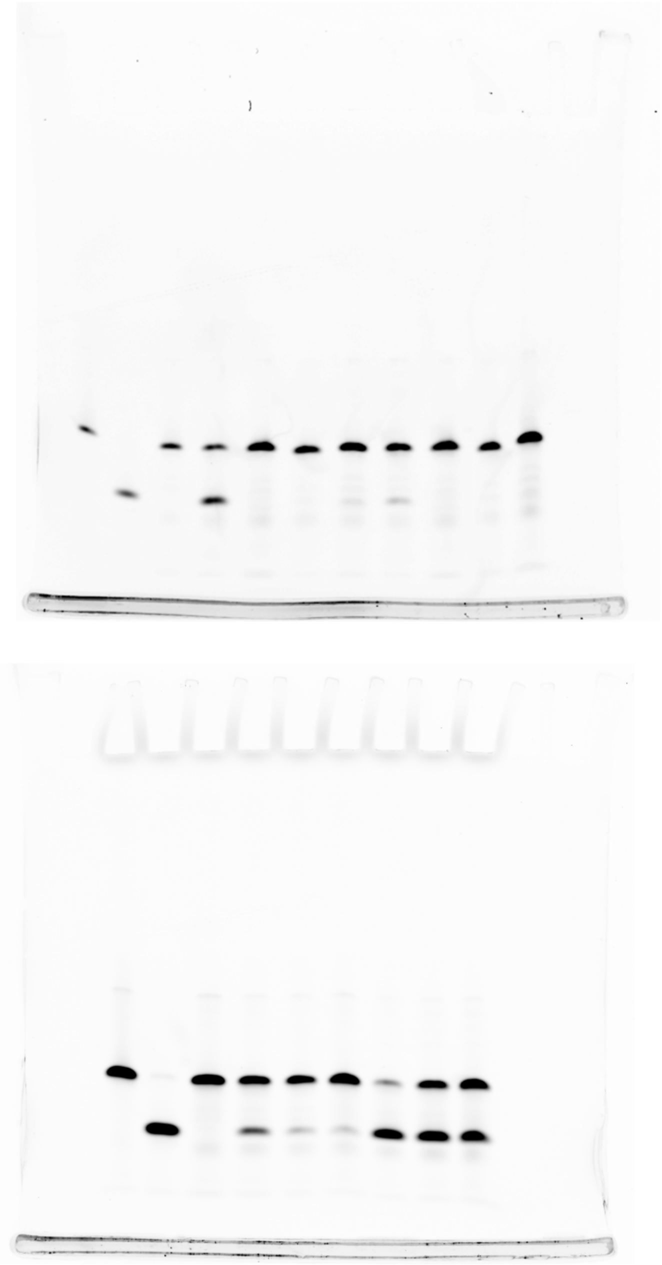

Supplement: Supplementary file 1 — Supplementary Fig. 1 and associated uncropped gel images. [file 41594_2023_1034_MOESM1_ESM.pdf]
