# Supplementary figures and images for: Structural basis of sequence-specific cytosine deamination by double-stranded DNA deaminase toxin DddA
Source: Nat Struct Mol Biol. 2023 Jul 17;30(8):1153–9. doi: 10.1038/s41594-023-01034-3 (PMC10442228; doi:10.1038/s41594-023-01034-3)

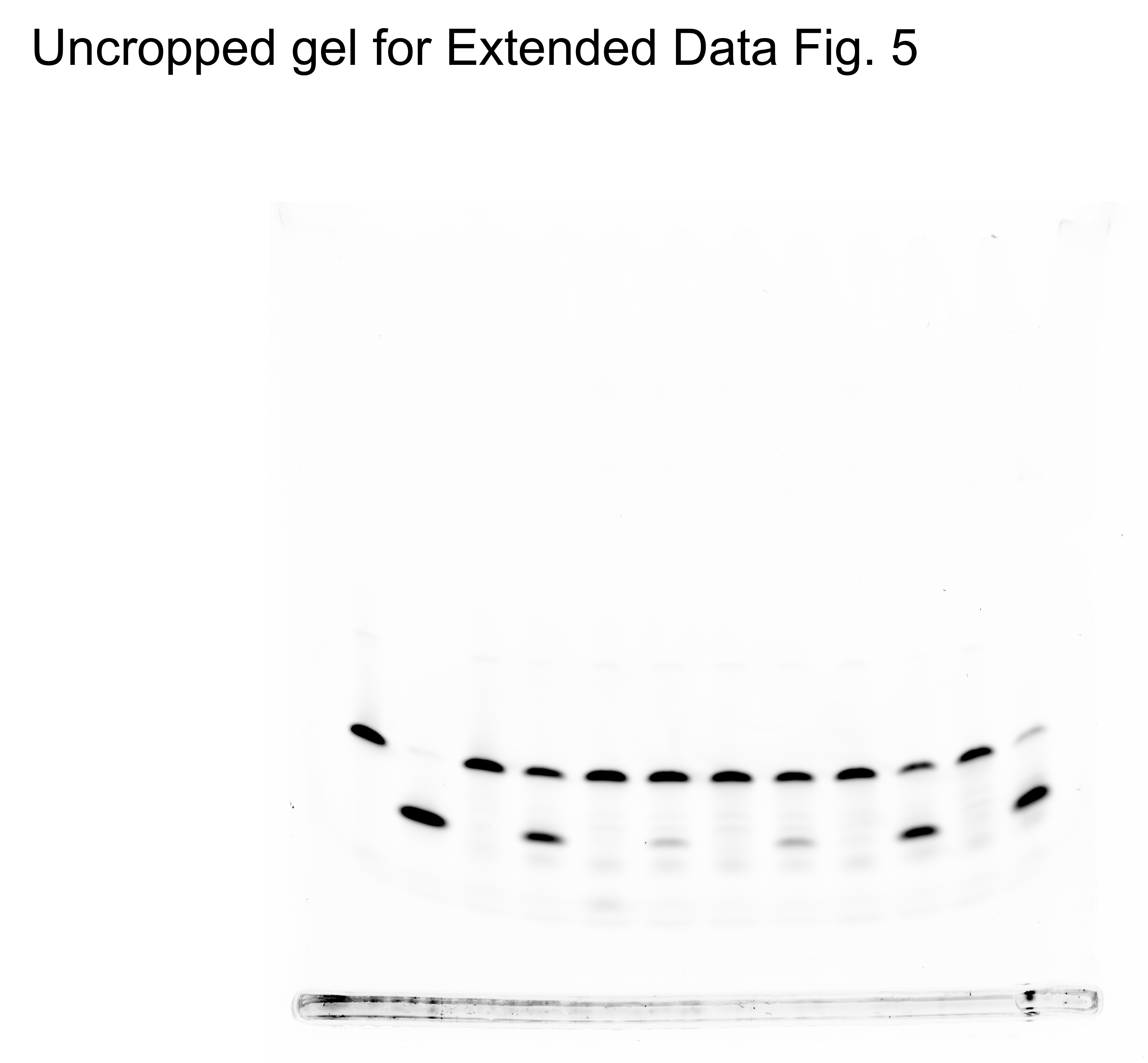

Supplement: Source Data Extended Data Fig. 5 — Uncropped gels. [file 41594_2023_1034_MOESM6_ESM.jpg]

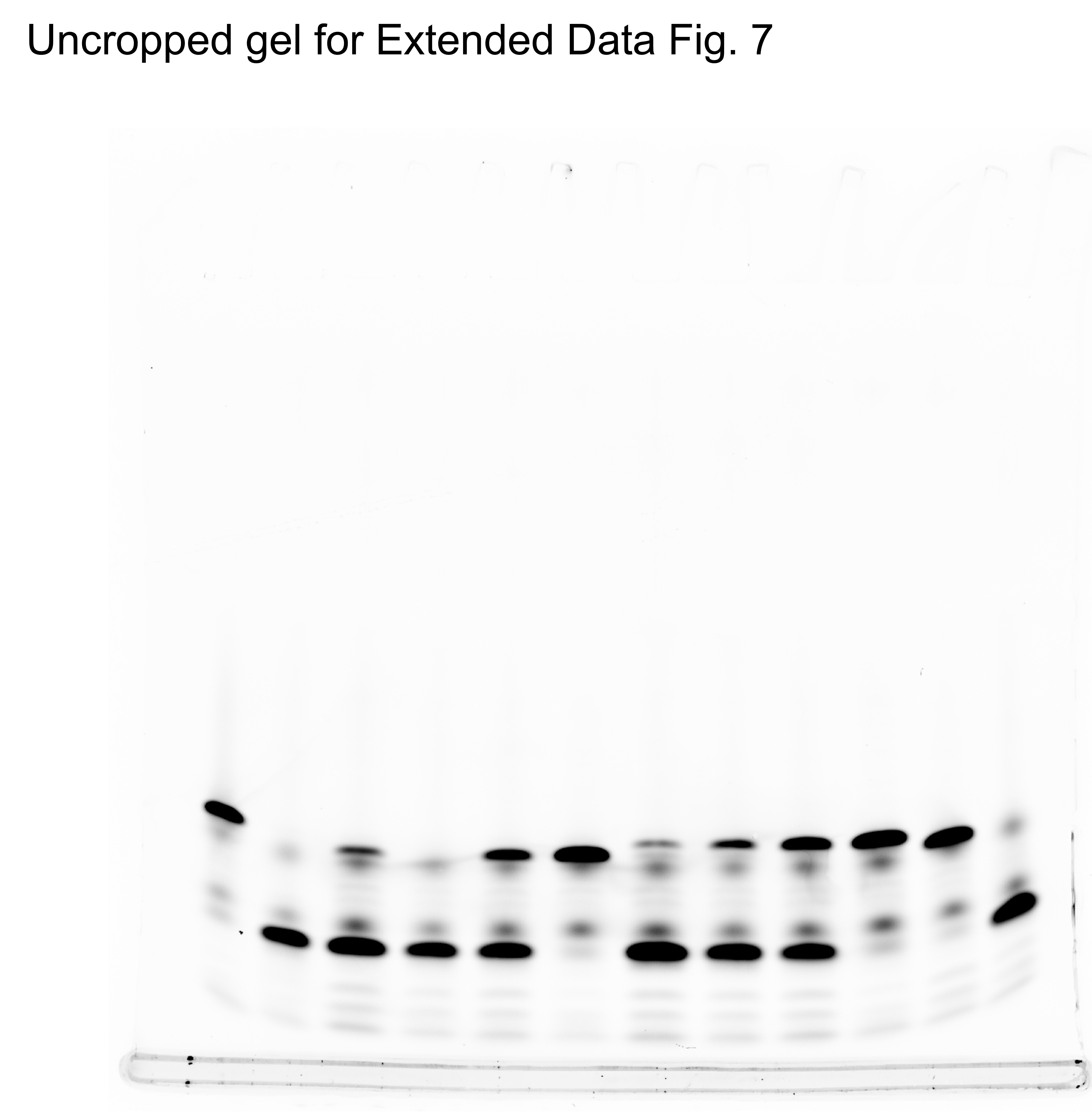

Supplement: Source Data Extended Data Fig. 7 — Uncropped gels. [file 41594_2023_1034_MOESM7_ESM.jpg]
